# Forward Programming of Cardiac Stem Cells by Homogeneous Transduction with *MYOCD* plus *TBX5*

**DOI:** 10.1371/journal.pone.0125384

**Published:** 2015-06-05

**Authors:** Elisa Belian, Michela Noseda, Marta S. Abreu Paiva, Thomas Leja, Robert Sampson, Michael D. Schneider

**Affiliations:** British Heart Foundation Centre of Research Excellence, National Heart and Lung Institute, Imperial College London, London W14 0NN, United Kingdom; New York Medical College, UNITED STATES

## Abstract

Adult cardiac stem cells (CSCs) express many endogenous cardiogenic transcription factors including members of the Gata, Hand, Mef2, and T-box family. Unlike its DNA-binding targets, Myocardin (Myocd)—a co-activator not only for serum response factor, but also for Gata4 and Tbx5—is not expressed in CSCs. We hypothesised that its absence was a limiting factor for reprogramming. Here, we sought to investigate the susceptibility of adult mouse Sca1^+^ side population CSCs to reprogramming by supplementing the triad of *GATA4*, *MEF2C*, and *TBX5* (GMT), and more specifically by testing the effect of the missing co-activator, Myocd. Exogenous factors were expressed via doxycycline-inducible lentiviral vectors in various combinations. High throughput quantitative RT-PCR was used to test expression of 29 cardiac lineage markers two weeks post-induction. GMT induced more than half the analysed cardiac transcripts. However, no protein was detected for the induced sarcomeric genes Actc1, Myh6, and Myl2. Adding *MYOCD* to GMT affected only slightly the breadth and level of gene induction, but, importantly, triggered expression of all three proteins examined (α-cardiac actin, atrial natriuretic peptide, sarcomeric myosin heavy chains). *MYOCD* + *TBX* was the most effective pairwise combination in this system. In clonal derivatives homogenously expressing *MYOCD* + *TBX* at high levels, 93% of cardiac transcripts were up-regulated and all five proteins tested were visualized. In summary: (1) GMT induced cardiac genes in CSCs, but not cardiac proteins under the conditions used. (2) Complementing GMT with *MYOCD* induced cardiac protein expression, indicating a more complete cardiac differentiation program. (3) Homogeneous transduction with *MYOCD* + *TBX5* facilitated the identification of differentiating cells and the validation of this combinatorial reprogramming strategy. Together, these results highlight the pivotal importance of *MYOCD* in driving CSCs toward a cardiac muscle fate.

## Introduction

The evolution of the heart from a simple contractile heart tube in certain invertebrates such as *Drosophila* to the complex multi-chambered organ of mammals relied on a conserved network of cardiac transcription factors as well as complex signalling pathways. The network of core cardiac transcription factors that regulates cardiac development includes members of the GATA family, such as Gata4; the HAND family, such as Hand1, -2; the LIM/homeodomain family, such as Isl-1; the MEF2 family, such as Mef2c; the NK-2 homeodomain family, such as Nkx2-5 and the TBX family, such as Tbx2, -5, and -20 [1–3]. Additionally, other transcription factors that are usually not classified as part of the core cardiac transcription factor network including serum response factor (SRF) [4] as well as its co-activator Myocardin (Myocd) [5] play crucial roles in guiding cardiogenesis. Cardiac transcription factors guide cardiac cell fate and lineage decisions in the embryo by regulating expression of cardiomyocyte-specific genes by binding to conserved DNA sequences in the promoter/enhancer regions of these genes.

The discovery that a single transcription factor can induce transition of a differentiated somatic cell into another cell fate was made as early as 1987, when expression of the transcription factor MyoD was demonstrated to convert fibroblast cell lines into stable skeletal myoblasts [6]. Ground-breaking studies of the last decade have demonstrated the transcription factor-induced conversion of various mature cell types into other mature cell types [7] as well as the generation of induced pluripotent stem cells (iPSCs) from fibroblasts by ectopic expression of four stem cell-enriched transcription factors Oct4, Sox2, Klf4, and c-Myc [8]. These discoveries overthrew the general view that development proceeds unidirectionally, and suggested that in fact it might be possible to use one or multiple transcription factor(s) to convert non-cardiomyocytes into cardiomyocytes, which has been achieved in multiple instances (reviewed in [9]). Among the first factors used for induction of cardiac differentiation are the core cardiac transcription factors Gata4, Mef2c, and Tbx5 (GMT), shown to transdifferentiate cardiac fibroblasts into cardiomyocytes in the absence [9,10] or presence of Hand2 [11] as well as the chromatin remodeling factor Baf60c, demonstrated to induce cardiac differentiation in embryonic non-cardiogenic mesoderm [12]. Other combinations of transcription factors were identified to reprogram non-myocytes into cardiomyocyte-like cells (GMT + Nkx2-5 [13]), many including the co-activator Myocardin (Myocd): MT + Myocd [14], GT + Nkx2-5 + Myocd [15], GMT + Myocd + SRF ± Mesp1 and Baf60c [16]. These contrasting results indicate that the selection of transcription factors to drive cardiac transdifferentiation may be further refined and that the cell type used, vectors carrying the factors, cell culture conditions, and reporter system all affect the outcome of the screen.

To date several populations of cardiac non-myocytes have been identified in the adult heart, termed cardiac progenitor or stem cells (CSCs), with the potential to form cells of the cardiovascular lineages at least in vivo, without ectopic transcription factors [17–22]. CSCs are a promising target cell population for cardiac regenerative therapy, aiming at replacing lost cardiomyocytes after cardiac injury, as a cell therapy product or target for activation in situ [23]. The reported CSC populations in the adult mammalian heart are founded upon numerous criteria that overlap incompletely, including surface markers [17,18], transcription factors [19], the side population (SP) dye efflux phenotype [24,25], the re-activation of epicardial markers denoting their developmental origin [21], and growth in tissue culture in colony-forming assays [22] or as “cardiospheres” [26]. The importance of the orphan receptor stem cell antigen-1 (Sca1) for cardiac regeneration and repair is suggested by studies of Sca1 knockout mice, which show a reduced number or function of resident CSCs, baseline contractile defects, and impaired responses to injury [27,28], similar to the essential roles of Sca1 demonstrated in skeletal muscle [29,30]. Moreover, endogenous Sca1+ cells contribute notably to cardiomyocyte creation after injury, as demonstrated by fate-mapping [31]. Exogenous Sca1^+^ CSCs differentiate into cardiomyocytes *in vivo* upon grafting into infarcted mouse hearts [18], as does the minuscule SP fraction of these cells [25], which is highly enhanced for clonal growth [24,32] and resembles in part the more primitive cardiac colony-forming unit-fibroblast (cCFU-F) [22], Currently, our understanding of the mechanisms and factors that control CSC differentiation is very limited. Thus, CSCs are an auspicious and relevant target population in which to study the mechanisms underlying progression toward a cardiomyocyte fate.

A widely observed characteristic of CSC populations is the expression of certain cardiac transcription factors, such as Gata4, Mef2c, Nkx2.5, Tbx5 and the second heart field marker LIM/homeodomain transcription factor Isl-1, in the absence of cardiomyocyte markers [17,18,20–22,33]. Gene expression profiling of cardiac Sca-1^+^ SP cells showed that they express progenitor markers and cardiac transcription factors, but lack expression of primitive mesodermal genes as well as later genes characteristic of mature cardiovascular cells. This molecular signature resembles a forme fruste (incomplete form) of cardiac mesoderm [32]. Moreover, Consistent with this interpretation, cardiac Sca-1^+^ SP cells largely derive from Mesp1-, Isl1-, Nkx2-5 precursors [32]. If the Gata, Mef2, and T-box families expressed at baseline are ordinarily sufficient to activate the differentiation program, the fact that CSCs remain in an undifferentiated state despite expression of an endogenic cardiogenic “cocktail” poses a potential paradox.

The present study was aimed at investigating if the baseline expression of cardiac transcription factors in CSCs predisposes them to undergo cardiac differentiation and, further, which minimal set of supplemental factors is required. An inducible lentiviral-mediated overexpression system was utilized to induce ectopic, drug-dependent, cardiac transcription factor expression in Sca1^+^ SP^+^ CSCs, and a tailored screen for induction of cardiomyocyte genes and proteins was performed to determine molecular differentiation. The previously identified combination of GATA4, MEF2C and TBX5 [9,10] activated a third of the analysed cardiac genes in CSCs, but none of the analysed proteins. Addition of MYOCD increased the number of activated cardiac transcripts and provoked readily detectable expression of all three cardiac proteins. The combination of MYOCD plus TBX5 was identified as the minimal combination required to induce cardiac differentiation markers at the RNA and protein level. On the basis of these findings, ectopic MYOCD and TBX5 then were homogenously expressed in CSCs, inducing 93% of the analysed cardiac transcripts, as well as all five cardiac proteins tested.

## Results

### Sca1^+^ SP^+^ CSCs express most cardiac transcription factors but lack Myocd

As a first step we determined which cardiac transcription factors, co-factors, and target genes are expressed in the CSCs for this study, using TaqMan Gene Expression Assays. We analysed 11 cardiac transcription factors including two members of the GATA-binding protein family (*Gata4*, *Gata6*), two members of the heart and neural crest derivatives expressed transcript family (*Hand1*, *Hand2*;), the LIM/homeodomain family member *Isl1*, two members of the myocyte enhancer factor family (*Mef2a*, *Mef2c*), the NK-2 homeodomain factor *Nkx2-5*; serum response factor (*Srf*), and two T-box (Tbx) family members (*Tbx5*, *Tbx20*), We also examined two cardiac-restricted co-factors, Baf60c and Myocd, which are expressed during cardiac development [5,34,35] and induce cardiac gene expression synergistically with the GMT factors Gata4, Mef2c and Tbx5 [12,36–39].

**Fig 1:**
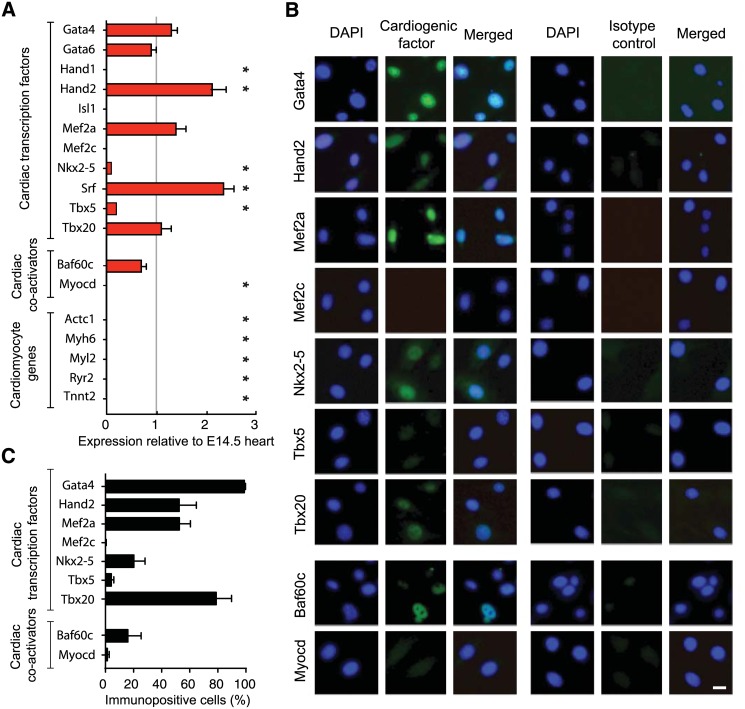
Cardiac transcription factor, co-activator and differentiation marker expression in adult mouse CSCs compared to the embryonic heart. Here and throughout, Lin^-^ / Sca1^+^ / SP^+^ CSCs were isolated from adult mouse myocardium and expanded for five passages before use. A: QRT-PCR. mRNA levels in CSCs (red) are expressed relative to the E14.5 mouse embryonic heart and are the mean ± SD of three independent experiments. *, p < 0.05 for CSCs versus embryonic heart. B, C: Immunocytocvhemistry. B: CSCs were cultured to subconfluent density and fixed for immunostaining. The transcription factors Gata4, Hand2, Mef2a, Nkx2-5, Tbx5, Tbx20 and chromatin remodeling protein Baf60c were present as nuclear-localized proteins, whereas neither Mef2c nor Myocd was detected. Factor-specific primary antibody binding was visualized using species-specific secondary antibodies conjugated with Alexa 488. Isotype control primary anitibodies were used as the control. Nuclei were counter-stained with DAPI. Bar, 20 μm. C: Quantitation of immunostaining by automated high-content image analysis. Data are the mean ± SD of three wells (≥ 2500 cells counted per well for each protein) and were concordant with the QRT-PCR profile for all nine factors tested by both methods.

CSCs expressed most of the analysed cardiac transcription factors and co-activators, with *Nkx2-5* and *Tbx5* at only low levels relative to others and omitting *Hand1*, *Isl1*, *Mef2c*, and *Myocd*, (Fig 1A). None of the five cardiac differentiation markers was detected. These results in freshly isolated CSC pools correspond closely to the conclusions obtained using cloned CSCs or single-cell expression profiles [32]. Compared to the embryonic mouse heart (E14.5), six of the cardiogenic factors (*Gata4*, *Gata6*, *Hand2*, *Mef2a*, *Tbx20* and *Baf60fc*) were expressed at comparable or even higher levels.

Immunostaining for cardiac transcription factors and co-activators showed that Gata4, Hand2, Mef2a, and Tbx20 were prevalent (50–98%) and appropriately nuclear-localized in CSCs, while Nkx2-5, Tbx5, and Baf60c were observed more sporadically (<20%; Fig 1B and 1C). The specificity of all antibodies was confirmed by staining of transfected versus control 293FT cells (**Panel A of** S1 Fig), and by Western blotting for all cases where detecting the denatured protein was workable (**Panel B of** S1 Fig). In agreement with the absence of *Mef2c* and *Myocd* RNA, these were not visualized at the protein level. With regard to reported essential factors for cardiac reprogramming, the absence of endogenous Mef2c [9,10] and Myocd [14,16,40,41] in the analysed CSCs stands out notably.

### Overexpression of a combination of 4 cardiogenic factors induces cardiac differentiation marker expression in CSCs

**Fig 2:**
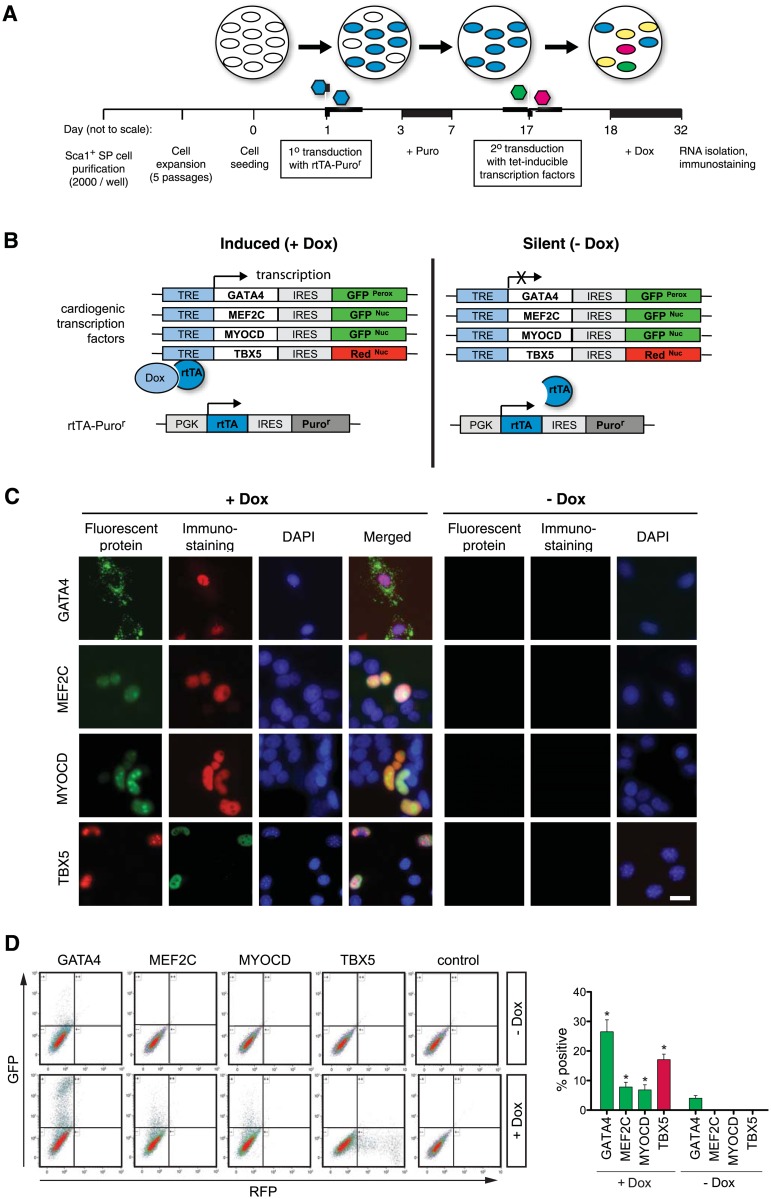
Dox-dependent expression of exogenous transcription factors in adult mouse CSCs. A: Timeline for the cardiogenic differentiation assays. CSCs were seeded in 6-well dishes (day 0), transduced with the rtTA-Puro^R^ lentiviral vector (day 1), and subjected to selection of successfully transduced cells in Puro (days 3–17). The selected transduced CSCs were re-seeded, transduced with Dox-inducible vectors for co-expression of transcription factors plus fluorescent reporters (day 18), and treated with Dox to induce the respective ectopic proteins (days 19–33). CSCs were then processed for RNA isolation and immunocytochemistry. B: Schematic representaiton of the induced (+Dox) and silent (-Dox) state. The system involves paired lentiviral vectors, rtTA-Puro^R^ constitutively expressing the reverse Tet transactivator (rtTA) and a Puromycin resistance gene (Puro^R^), and a Dox-inducible series encoding the respective cardiac transcription factors and targeted reporter proteins via a Tet-responsive element (TRE). Co-expression in these bicistronic vectors is mediated by an internal ribosome entry site (IRES). C: Immunocytochemistry showing Dox-dependent expression of the ectopic transcription factors in transfected 293FT cells, which lack the corresponding endogenous factors, and appropriate intracellular targeting of all the respective reporters. D, E: Flow cytometry showing Induction of the transcription factor-reporter cassettes in transduced CSCs (MOI = 100). D: Representative dot plots. E: The percentage of fluorescent protein-expressing CSCs is shown as the mean ± SD for three samples, and is representative of additional experiments using one or more of the indicated factors. *, p < 0.05 for the presence or absence of Dox.

**Fig 3:**
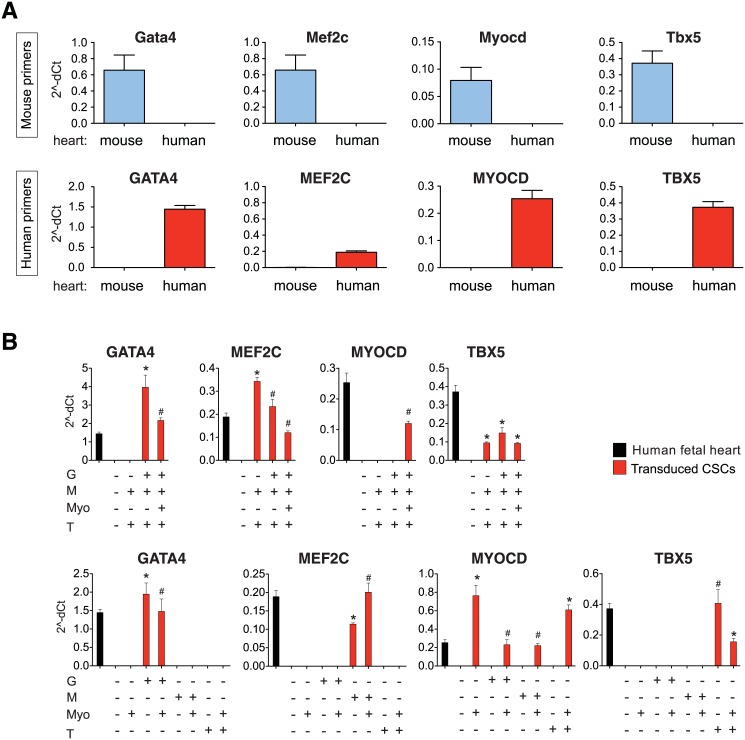
Species-specific detection of ectopic cardiac transcription factors in CSCs. A: Validation of specificity was confirmed using mouse versus human TaqMan Gene Expression Assays to detect the indicated endogenous factors in the E14 embryonic mouse heart (blue) and 12-week human fetal heart (red). Data are the mean ± SD of three independent experiments. B: Human factor expression in adult mouse CSCs. Transduced cells were treated with Dox for 14 days. Upper low, results comparing transduction of MT, GMT, and MyoGMT; lower row, results comparing single transduction of the GMT factors versus co-transduction with *MYOCD*. Expression was typically decreased as the number of co-transducing viruses increased. *, p < 0.05 for the treated CSCs versus human fetal heart, for reference.

**Fig 4:**
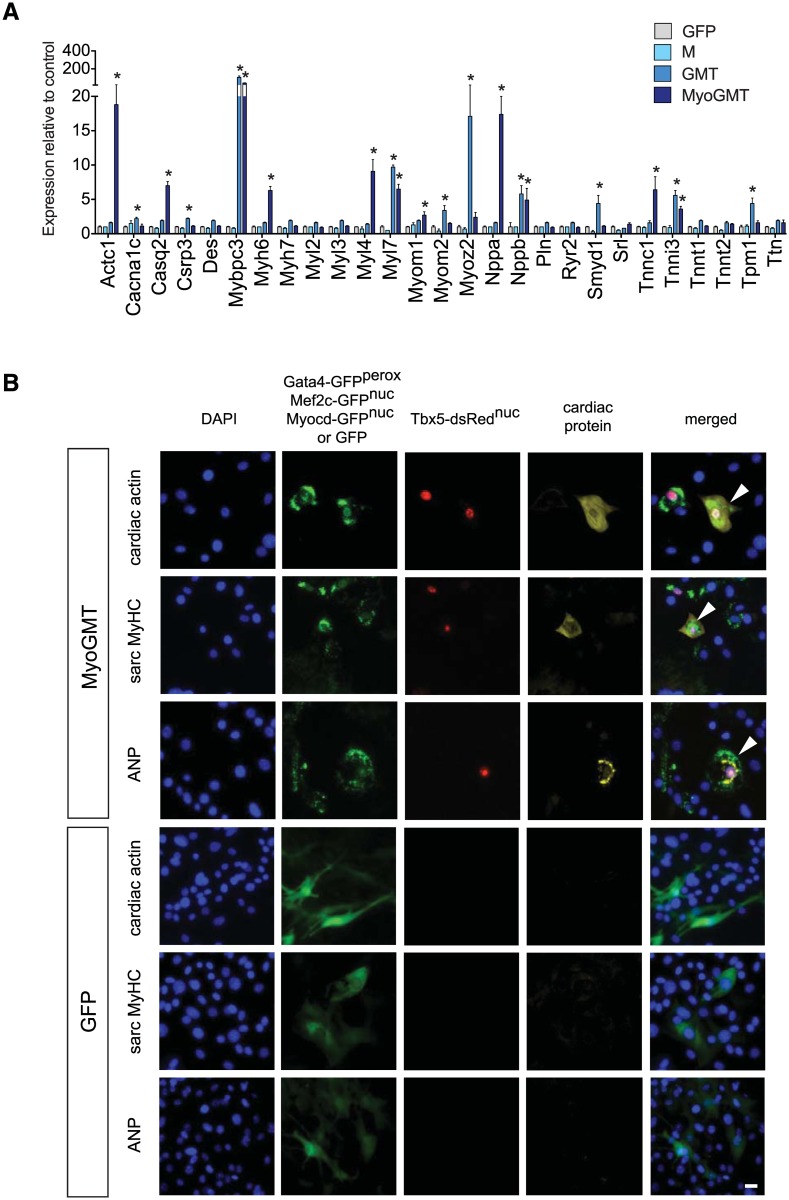
Cardiac gene and protein induction in CSCs by MyoGMT. A: QRT-PCR comparing MEF2C alone (the GMT factor absent from the CSCs), the GMT triad, and the 4-factor combination of MyoGMT. Data are the mean ± SD of three independent experiments. *, p <0.05, for factor-transduced versus GFP-transduced CSCs. Results here and in Fig 5 are taken from the identical three experiments, separated for clarity of the respective comparisons. B: Immunocytochemistry showing induction of the indicated cardiac proteins in CSCs by MyoGMT. Cells expressing the indicated proteins were identified using secondary antibodies conjugated with Alexa 647 (pseudocoloured yellow for enhanced visibility). Co-expression of GFP^perox^ + GFP^nuc^ + dsRed^nuc^ (expressed with the ectopic factors G, Myo / M and T, respectively), was assessed to identify the successfully co-transduced cells. No induction was detected in cells transduced with the GFP control vector (lower panels), or cells transduced with M and GMT in the absence of *MYOCD* (not shown). Similar results were observed in each of two independent experiments.

We postulated that the absence of Mef2c, Myocd or both might be a mechanism by which adult CSCs are stably maintained in a progenitor state whose gene expression profile otherwise largely resembles the cardiac mesoderm seen transiently in native embryos and differentiating pluripotent cells. Alternatively, or in addition, one or more of the expressed factors might be insufficient, at its native level of expression. To investigate these hypotheses, we overexpressed key cardiac transcription factors or their co-factor Myocd in Sca1^+^ SP^+^ CSCs using a doxycycline (Dox)-inducible lentiviral system (Fig 2A) enabling temporally controlled induction and repression of the exogenous cardiac transcription factors [42,43]. The system consists of a vector encoding the reverse tetracycline transactivator (rtTA) [42] and a series of vectors encoding the inducible cardiac transcription factor genes downstream of the tetracycline-responsive element (TRE) (Fig 2B). The rtTA-encoding vector also encodes a puromycin resistance cassette to enable selection of successfully transduced cells. In the absence of Dox, rtTA is unable to bind the TRE and the cardiac transcription factor is not transcribed. However, when Dox is added it interacts with the rtTA, which consequently binds to the TRE and induces the transgene. Four lentiviral constructs were tested, encoding the triad of GATA4, MEF2C and TBX5 plus the co-activator MYOCD (Fig 2B). A further feature of the lentiviral backbone utilised is co-expression of the cardiogenic gene plus a nuclear- or peroxisomal-localised fluorescent protein via an internal ribosome entry site (IRES), simplifying the detection of Dox-dependent transgene induction. Dox-dependent induction of the cardiogenic proteins was confirmed by immunostaining in 293FT cells and was concordant with the respective reporter proteins (Fig 2C; see also S1 Fig). The prevalence of transduction was maximal at a multiplicity of infection (MOI) of 100, and the concentration of Dox saturating at 500–1000 ng/ml (S2 Fig). Dox-dependent control was then confirmed in CSCs themselves, using flow cytometry (Fig 2D). Here and in Figs 3 and 4, Sca1^+^ SP^+^ CSCs were isolated from adult mouse hearts, purified by preparative flow sorting (roughly 10,000 per heart), expanded for five passages in growth medium, transduced with lentiviral constructs, and then treated with Dox for 14 days to activate the exogenous factors.

Because two of the cardiogenic genes to be tested are already expressed endogenously in CSCs (*Gata4*, *Tbx5*), we used their highly conserved human orthologues for overexpression in this mouse background, thus enabling us to track the exogenous human transgenes versus their endogenous mouse counterparts (Fig 3). The QRT-PCR probes were confirmed as stringently species-specific (Fig 3A), even though conservation at the amino acid level was 100% for the DNA-binding and dimerization domains, 93–100% in regions for predicted factor-factor interactions, and 86–98% in the transcriptional activation domains. Using the human-specific QRT-PCR assays, expression of the ectopic human genes was confirmed for each sample, achieving levels comparable to or exceeding these genes’ expression in the human fetal heart (Fig 3B).

Despite the presence of endogenous Gata4 and Tbx5 in the CSCs, addition of *MEF2C* did not induce cardiac differentiation markers, under the conditions used (Fig 4). Even the full triad of *GATA4*, *MEF2C* and *TBX5* (GMT) induced only a minority of the cardiac transcripts (10 of 27; Fig 4A). Most of these genes were up-regulated 5- to 20-fold, by QRT-PCR, relative to control CSCs, yet no induction of was detected for any of the three cardiac proteins tested: atrial natriuretic peptide (ANP), α-cardiac actin, and sarcomeric myosin heavy chains (sarc MyHC) (data not shown). Thus, GMT was insufficient to induce cardiac differentiation in CSCs, in agreement with observations made by most investigators in fibroblastic cells including cardiac fibroblasts [11,44].

Importantly, whereas co-expression of *MYOCD* with GMT (MyoGMT) had only a small further effect on the range of RNAs induced (11 of 27 analysed; Fig 4A), all three proteins tested—ANP, α-cardiac actin, and sarc MyHC—were readily visualized by immunocytochemistry(Fig 4B). *MYOCD/MEF2C* + *GATA4* + *TBX5* transduced cells were identified, respectively, by co-expression of GFP^nuc^ + GFP^perox^ + dsRed^nuc^ encoded by the bicistronic lentiviral constructs. Because the corresponding three transcripts were induced exclusively here with MyoGMT, the results for immunostaining more likely reflect transcriptional synergy than translational control. Induction of the cardiac proteins was seen exclusively in transduced cells co-expressing all three reporters, confirming three- or four-way transduction with the cardiogenic genes. in this sub-population, the prevalence of induction was 2–3% for each of the three target proteinss. Most of the induced transcripts, compared to the day 14.5 embryonic heart, were at least 10-fold lower in the transduced CSCs (data not shown), ascribable at least in part to the low efficiency for co-transduction with four factors and, potentially, to promoter competition. No up-regulation of cardiac RNAs or proteins was observed in control CSCs, indicating lack of any differentiation as a mere consequence of cell culture, the retroviral backbone, or Dox administration (Fig 4B).

Thus, while supplementation with *MEF2C* alone or the GMT triad was not sufficient to induce cardiac differentiation in CSCs under these conditions, addition of *MYOCD* to GMT initiated the expression of several essential cardiomyocyte proteins.

### Identification of *MYOCD* and *TBX5* as a minimal cardiogenic combination

**Fig 5:**
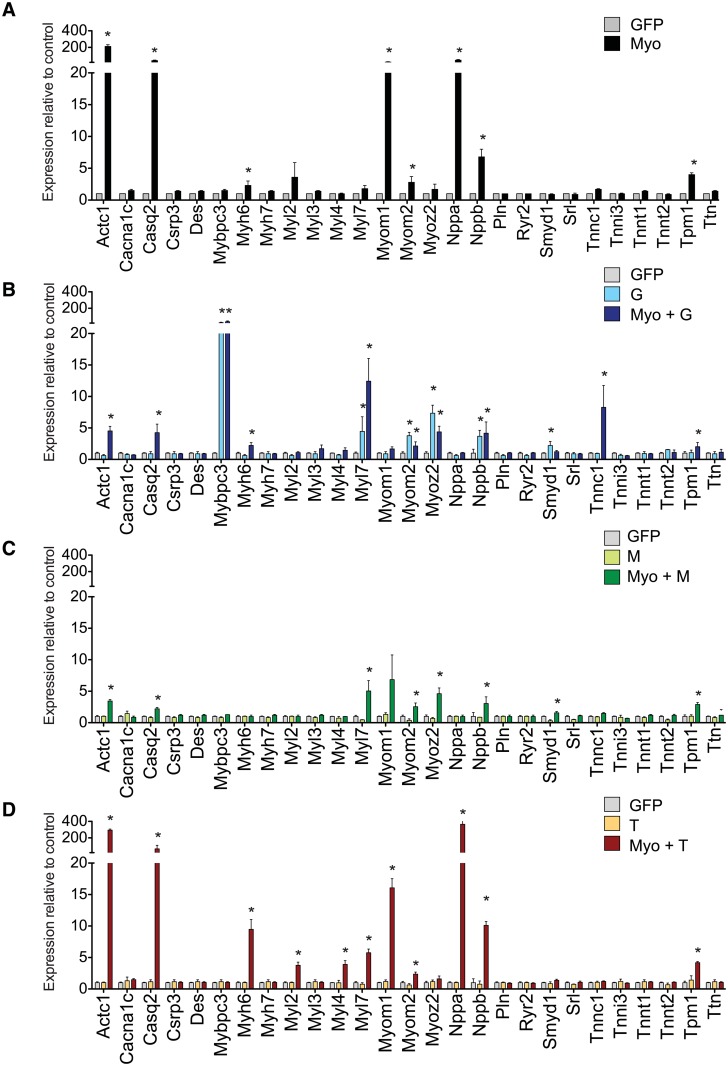
Cardiac gene induction in CSCs by single GMT factors ± *MYOCD*. QRT-PCR comparing *MYOCD* alone with *GATA4*, *MEF2C*, and *TBX5* in the absence or presence of *MYOCD*. Data are the mean ± SD of three independent experiments. *, p <0.05, versus the GFP control.

The combination of *MYOCD* plus three transcription factors GATA4, MEF2C and TBX5 induces partial cardiac differentiation in CSCs, but the contribution of individual factors to this response was unknown. To identify a minimal cardiogenic combination for CSCs, cell were transduced with single factors in the absence or presence of MYOCD and ectopic factor expression was induced by Dox for two weeks. By QRT-PCR, *MYOCD* alone induced 9 of 27 analysed cardiac markers (33%; Fig 5A), confirming previous evidence for the cardiogenic potential of *Myocd* in some settings [5,36,45]. In contrast, neither *MEF2C* nor *TBX5* alone induced any cardiac gene’s expression, and GATA4 alone induced 6 of the markers analysed (22%; Fig 5B–5D). Thus, when overexpressed as single factors, only *MYOCD* and *GATA4* had cardiogenic potential in CSCs.

Next, *MYOCD* was tested in combination with each transcription factor singly (Fig 5). Overall, *MYOCD* + *TBX5* (MyoT) was most potent combination, inducing 11 out of 27 cardiac genes (41%) of the analysed cardiac markers. Compared to *MYOCD* alone, addition of *TBX5* induced 6 genes to significantly higher levels (*Actc1*, *Myh6*, *Myl4*, *Myl7*, *Nppa*, *Nppb*) and 1 to lower levels (*Tnnc1*) (p < 0.01) (Fig 5D). Unlike this synergistic effect of *TBX5*, exogenous *GATA4* and *MEF2C* each inhibited the *MYOCD*-induced expression of cardiac genes. Addition of *GATA4* to *MYOCD* (MyoG) significantly reduced 10 genes (*Actc1*, *Cacna1c*, *Casq2*, *Csrp3*, *Mybpc3*, *Myh7*, *Myom1*, *Nppa*, *Tnni3*, *Tnnt1*) and further induced only 2 (*Mybpc3*, *Myl7*) (p < 0.01) (Fig 5B). Addition of *MEF2C* to *MYOCD* (MyoM) was similarly ineffective, reducing 4 genes while up-regulating just Smyd1 (p < 0.01) (Fig 5C).

Thus, in marked contrast to broad inhibitory effects of *GATA4* and *MEF2C* on the *MYOCD*-induced cardiac program, *TBX5* exhibited strong synergy with *MYOCD*. Hence, co-expression of *MYOCD* and *TBX5* was identified for further testing as the most potent pairwise combination.

### Homogeneous expression of *MYOCD* and *TBX5* induces highly effective cardiac transcript and protein expression in CSCs

**Fig 6:**
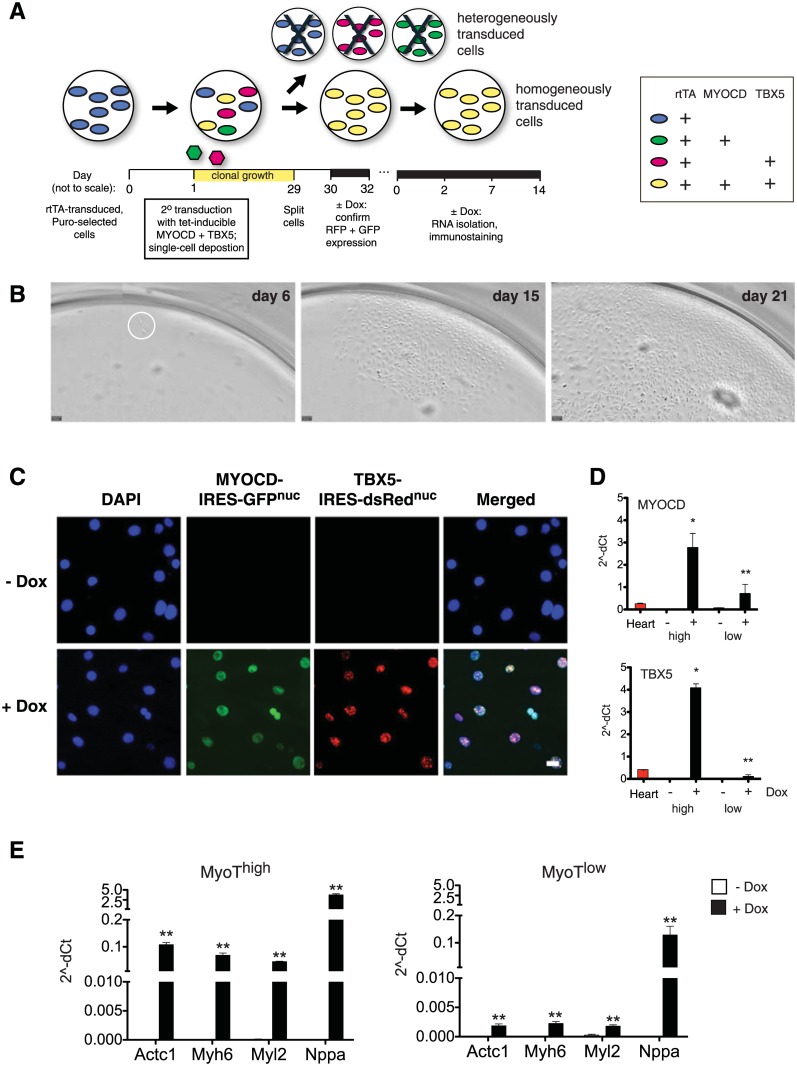
Homogeneous transduction of CSCs with *MYOCD* and *TBX5*. A: Timeline of the protocol, highlighting the use of rtTA-Puro^r^ CSCs and secondary transduction with Dox-dependent *MYOCD* + TBX5, as in Fig 2A. For homogeneous transduction, single-cell deposition was performed using a preparative cell sorter (day 1), clonal growth was permitted for four weeks, the single-cell derivatives were passaged (day 29), and the CSCs were then cultured ± Dox to confirm co-expression of the dual fluorescent reporters (days 30–32). Single-cell progeny expressing both were taken forward ± Dox for differentiation studies. B: Serial images of a representative single-cell clone at 6–21 days. C: Homogeneous co-expression of MYOCD-IRES-GFP^nuc^ and TBX5-IRES-dsRed^nuc^ in clonal CSCs engineered by the method in panel A. Note, by comparison, the marked heterogeneity of transduction in Fig 4B. D: QRT-PCR showing stringent Dox-dependent expression of *MYOCD* and *TBX5* in the homogeneously transduced cells. The two clones are designated MyoT high and MyoT low, respectively, in accordance with the elicited expression levels. *, p < 0.05, and **, p < 0.01 versus human fetal heart. E: Dose-dependent induction by MyoT at 14 days was confirmed for all four cardiac genes, tested as a prelude to full evaluation of the higher line in Figs 7 and 8. **, p < 0.01 for day 0 versus day 14.

To study the impact of co-expressed factors without the confounded dilutional effect of incompletely transduced cells, homogeneous transduced CSCs were engineered (Fig 6A). rtTA-transduced cells selected in puromycin were transduced with Dox-inducible MyoT as before, but were immediately subjected to single-cell deposition using a preparative cell sorter and were expanded for one month (Fig 6B). At that stage, cells were split and a portion of each culture was treated for two days in the absence or presence of Dox of to confirm dual induction of the Dox-dependent RFP and GFP reporter genes. In cultures expressing both reporters (10% of the secondary clones), >99% of the cells co-expressed *MYOCD*-IRES-GFP^nuc^ and and *TBX5*-IRES-dsRed^nuc^ (Fig 6C). Based on this, two homogeneously transduced populations were chosen for expansion in the absence of Dox and banked for further study, having high and low expression of the exogenous factors respectively (MyoT^high^, MyoT^low^; Fig 6D). Preliminary experiments confirmed a dose-dependent effect of MyoT at 14 days (Fig 6E), and on this basis the higher-expressing cells were more fully investigated using the complete panel and time-course.

**Fig 7:**
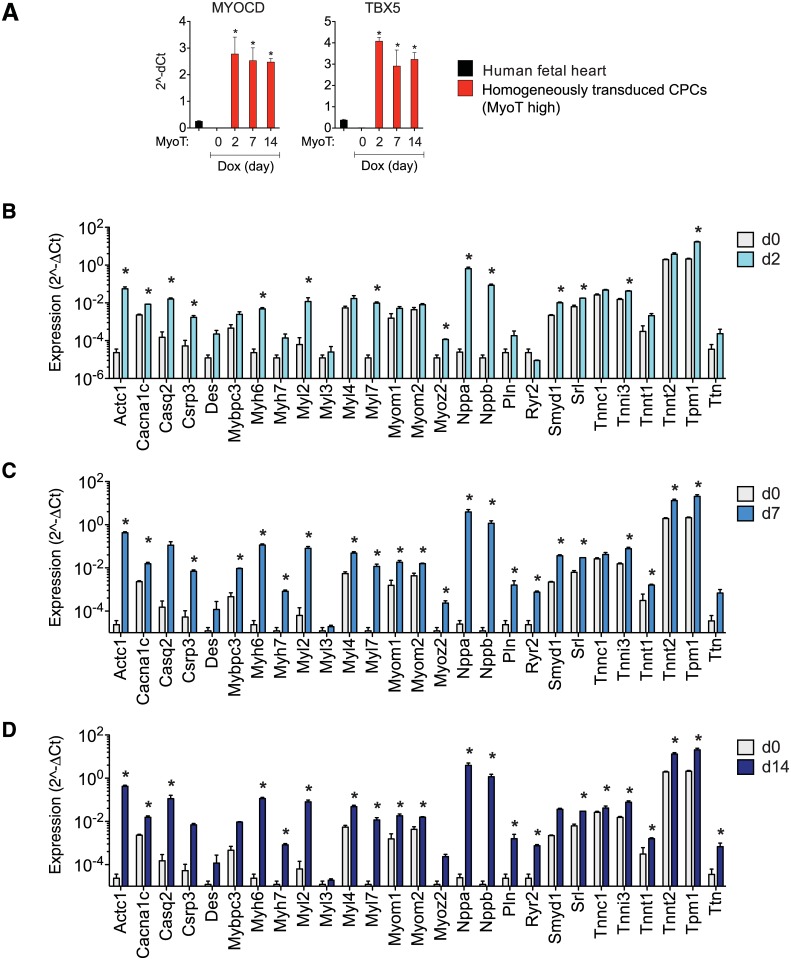
Cardiac gene induction in CSCs by homogeneous transduction with *MYOCD* and *TBX5*. A: QRT-PCR showing stable expression of exogenous cardiac transcription factors in CSCs transduced homogenously with MyoT. Levels of both factors were 2.5- to 4-fold higher than in human fetal heart. *, p < 0.05. Data are the mean ± SD of three independent experiments. B-D: QRT-PCR showing cardiac gene expression after Dox administration for 2, 7, and 14 days. Data are the mean ± SD of three independent experiments. *, p < 0.05 versus the GFP control.

**Fig 8:**
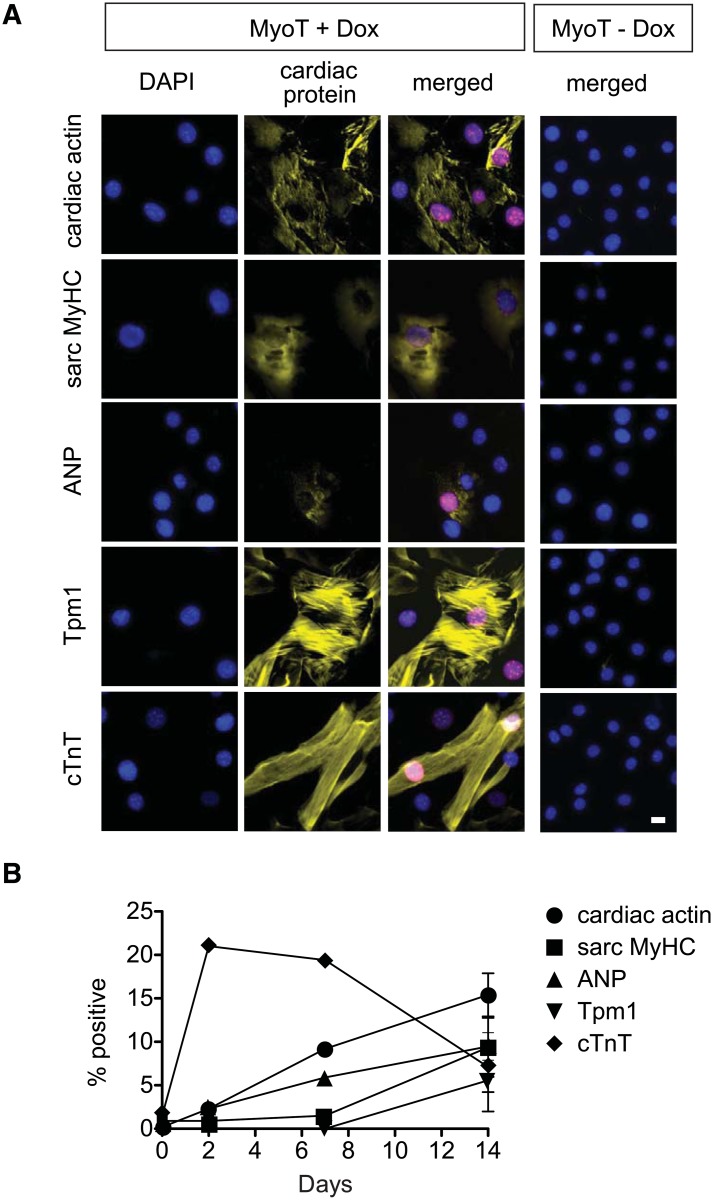
Cardiac protein induction in CSCs by homogeneous transduction with *MYOCD* and *TBX5*. CSCs transduced homogenously with MyoT were cultured for 14 days ± Dox, then were analyzed by immunocytochemistry as in Fig 4. A: Representative images. Bar, 20 μm. B: Prevalence of cardiac protein expression in the MYOCD-IRES-GFP^nuc^-positive, TBX5-IRES-dsRed^nuc^-positive cells. Three samples were analysed for each protein (≥ 1600 cells). Data are the mean ± SD of three independent experiments (14d) or are single experiments for 2 and 7d. p < 0.01 for all proteins at 14 versus 0 days. Bar, 20 μm.

Ectopic *MYOCD* and *TBX5* were stably maintained over the course of the experiment (Fig 7A). Even 2 days after induction of *MYOCD* and *TBX5*, 14 of the 27 cardiac markers were significantly higher than at day 0 (Fig 7B). This increased to 22 at 7 days (81%; all excepting *Casq2*, *Des*, *Myl3*, *Tnnc1*, *Ttn*; p < 0.05; Fig 7C). At 14 days, three of these five were induced (*Casq2*, *Tnnc1*, *Ttn*), whereas *Csrp3*, *Mybpc3*, *Myoz2*, and *Smyd1* no longer were significant (p < 0.05; Fig 7D). Overall, CSCs expressed the majority of cardiac marker progressively over the analysed time course of 2 weeks, with 25 out of 27 cardiac markers found to be induced after one or two weeks of ectopic *MYOCD* and *TBX5*. Notably, Induction at the protein level was substantiated for all five markers of differentiation tested (ANP, CaA, sarc MyHC, Tpm1, Tnnt2), detected in 2–20% of the homogeneous transduced CSCs (Fig 8), though sarcomere organization was not evoked on the conventional substratum used here. Most of the proteins’ prevalence increased monotonically over 2 weeks in culture. In contrast, cTnT was expressed in 20% of the cells at 2 days, and subsequently subsided to a percentage similar to that of the other cardiac differentiation markers. This highlights the importance of test an ensemble of markers over a sufficent time course to establish representative results. No cardiac proteins were expressed in the absence of Dox (Fig 8).

Given the cardiac differentiation observed in homogeneously transduced cells, we analysed if cardiac progenitor markers were down-regulated concomitantly, focusing on those most relevant to isolating the starting CSCs (*Abcb1* and *Abcg2*, which encode ATP-dependent drug transporters responsible for the SP phenotype, and *Ly6a*, which encodes Sca-1 itself). As postulated, *Abcb1*, *Abcg2* and *Ly6a* were down regulated 3- to 4-fold concurrent with cardiac differentiation induction, and were repressed as early as day 2, when cardiac markers first were expressed in this system (p < 0.05; **Panel A of** S3 Fig). Cardiac transcription factor expression was unchanged following ectopic *MYOCD* and *TBX5* expression for up to two weeks (**Panel B of** S3 Fig).

In conclusion, the induction of 93% of cardiac transcripts, the expression of all 5 tested markers as readily detectable proteins, and the repression of cognate CSC markers indicates a transition from the progenitor toward the cardiomyocyte stage induced by homogeneous transduction with *MYOCD* plus *TBX5*.

## Discussion

Expression of cardiac transcription factors associated with embryonic heart development is a common characteristic of most adult CSC populations described to date [17–19,21,32,33][32,46] The DNA-binding cardiac factors most abundant in Sca1^+^ SP^+^ CSCs were *Gata4*, *Gata6*, *Hand2*, *Mef2a*, *Srf* and *Tbx20*, whereas others, such as *Hand1*, *Isl1*, *Mef2c*, *Nkx2-5*, and *Tbx5* were present at low or undetectable levels. Our expression profiling of CSCs confirms the seeming paradox of cardiac transcription factor expression that might be expected to suffice for activation of cardiomyocyte-specific genes that are direct targets of these factors during cardiogenic differentiation [23]. Indeed, the cardiac transcription factors ordinarily expressed in CSCs are known to bind cardiac promoters and activate cardiac genes as evidenced by luciferase reporter gene assays [47–53], promoter binding site analysis [54,55] knock-down mice [56–58], and chromatin immunoprecipitation high-throughput sequencing combined with knock-down studies [59].

The repeatedly observed inability of endogenous cardiac transcription factors to activate their direct cardiomyocyte-specific targets in CSCs under baseline conditions prompted us to investigate if absent transcription factors or co-activators, in addition to the endogenous ones, were required to activate cardiac target genes. Given the large number of factors present already, we postulated that this might be achieved with just one or possibly two. In contrast to the initial reports of GMT inducing the transition of cardiac fibroblasts to cardiomyocyte-like cells [9,10,60], the results presented here show that GMT induced very incomplete differentiation in clonogenic CSCs [14,44]. This difference may not be due specifically to cell type, as a number of independent groups have contested the impact of GMT alone [14,44], and the stoichiometry among these genes affects both the prevalence and extent of differentiation [61]. Given the rarity of Sca1^+^ SP^+^ CSCs in adult myocardium, the cells investigated in the present report would presently seem unlikely to explain the yield of cardiomyocytes obtained with GMT or other cardiogenic cocktails in the intact heart [9–11]. Conversely, the expression of some cardiogenic factors in cardiac fibroblasts [62] may help explain the cells’ reported susceptibility of reprogramming toward a cardiomyocyte phenotype, compared to fibroblasts originating from the tail.

The striking result in the present study was the large cardiogenic effect achieved with *MYOCD* alone or *MYOCD* plus *TBX5*, which are absent and weakly expressed in the CSCs respectively. It is logical to infer that this distinctive phenotype might be due to the baseline expression of essential DNA-binding factors and the chromatin remodeling protein Baf60c in CSCs. However, presently, we cannot exclude the counter-hypothesis that steady-state expression of those factors is less instrumental than other features, such as, conceivably, a permissive epigenetic landscape in CSCs [63,64]. Preliminary studies have suggested that changes take place in the epigenetic signature of cardiomyocyte-specific genes during the GMT-induced transition of cardiac fibroblasts into the cardiac lineage [9]. The authors analysed the presence of trimethylated histone H3 of lysine 27 (H3K27me3) and lysine 4 (H3K4me3), which mark transcriptionally inactive or active chromatin [64], respectively, on the promoters of three cardiomyocyte-specific genes [9]. At baseline the fibroblasts harbored repressive H3K27me3 marks on the target gene promoters, but upon transduction with GMT and consequent induction of cardiac differentiation these methylation marks were depleted at the promoters of all the genes analysed, reaching levels comparable to those in cardiomyocytes [9]. Changes in the epigenetic status of cardiac gene promoters might be catalyzed by chromatin-remodeling factors recruited by the overexpressed ectopic cardiac transcription factors [34,65,66].

The putative stem cell nature of CSCs has been questioned due to the lack of fulfilling all the functional criteria for stemness originally established for the hematopoietic stem cell. For instance, c-kit^+^ CSCs make an unclear contribution to cardiac myogenesis in vivo [67], and hematopoietic stem cells do not require genetic reprogramming to display their full potential after bone marrow transplantation [68]. However, evidence exists both for a contribution of endogenous Sca1^+^ CSCs to cardiac self-repair [31] and for the multi-lineage potential of their single-cell progeny after grafting to the heart [32]. Despite the lack of any obvious other difference from Lin^-^/Sca1^+^/PDGFRa^+^/Non-SP cells, addition of the SP phenotype in Lin^-^/Sca1^+^/PDGFR α^+^ CSCs is associated with a 10-fold greater cloning efficiency [32]. However, it is not yet known whether the SP phenotype, which tracks with both long-term self-renewal and plasticity in bone marrow [68], contributes equivalently to CSCs’ plasticity and response to reprogramming factors.

From a technical standpoint, it is important to note that cardiac reprogramming studies have typically used heterogeneous populations containing singly transduced multiply transduced, and non-transduced cells [9,11,14,15,44], potentially limiting the procedures’ effect, and the co-transduction efficiency is unknown, preventing direct comparisons. We addressed this using bi-cistronic lentiviral vectors co-expressing the respective cardiac transcription factor along with a fluorescent protein having a cellular localization tag [15]. Cells successfully transduced with the GATA4-, MEF2C-, TBX5-, or MYOCD-encoding lentiviral vector co-express GFP^perox^, GFP^nuc^, dsRed^nuc^ or GFP^nuc^, respectively. In single viral vector transductions, the frequency of GATA4-, MEF2C- and TBX5-transduced cells was 47, 18 and 58%, respectively, indicating that the different factor-encoding lentiviral vectors vary in their efficiency to transduce SP cells, despite being utilised at the same MOI. Reasons for the vectors’ differences in transduction efficiency include potential cell death or impaired proliferation in cells overexpressing particular factors. The frequency of TBX5 and GATA4 co-expressing CSCs was 20%, while the frequency of TBX5 and MEF2C co-expressing cells was 10%. With the vectors used presently, the frequency of triple and quadruple transduced cells could not be determined using flow cytometry because GFP^perox^ and GFP^nuc^ cannot be distinguished by this method. However, the use of subcellular localised fluorescent proteins enabled us to identify triply transduced cells quickly and unambiguously by immunofluorescence microscopy and to focus on these in the quantitation of cardiac protein induction [15]. Future studies can address this problem with bicistronic vectors that encode a wider range of fluorescent proteins discernable by flow cytometry [69]. The limited number of co-transduced cells, here and in most published work on forward programming, could dilute not only the number of differentiating cells but also the cell-nonautonomous effects that promote differentiation, potentially including paracrine secreted proteins, cell-cell contact, nanotubular connections, and exosomes [70–73].

## Materials and Methods

All animal procedures were performed under UK Home Office approval (PL 70/6806, 70/7880) and local approval by the Imperial College London Institutional Animal Care and Use Committee. Mice were euthanised by cervical dislocation, a Schedule 1 method.

### Cells

To isolate cardiac Lin-/Sca1^+^/SP cells, ten hearts from adult male C57BL/6 mice (8–13 weeks old; Charles River) were minced with scissors then enzymatically dissociated in 10 ml of digestion buffer (Hank's buffered salt solution; 50 μg/ml DNAse I, Roche Applied Science; 100 μg/ml Liberase TH, Roche Applied Science; 10 mM HEPES, Life Technologies; 30 mM taurine, Sigma-Aldrich), for 5 cycles of 10 min at 37°C. After each round, undigested tissue was allowed to settle at the bottom of the tubes and the supernatant containing released cells was filtered through 70 μm nylon mesh (BD Falcon) to obtain a single cell suspension. To arrest the enzymatic activity, stopping buffer (Hank's buffered salt solution; 10 mM HEPES; 30 mM taurine; 20% fetal bovine serum [FBS], Thermo Scientific) was added to the filtered cell suspension (1:1 volume) after each round of digestion. Under these conditions, most adult cardiomyocytes are lysed (Oh et al. 2003). The resulting cardiomyocyte-depleted cell suspension was then depleted of cells expressing hematopoietic lineage markers (Lin) including CD5, CD45R, CD11b, Gr-1, 7–4, and Ter-119, using a cocktail of biotinylated antibodies and super-paramagnetic microbeads (Lineage Cell Depletion Kit, #130-090-858, Miltenyi Biotec). The labeled cell suspension was passed through a magnetic cell sorting (MACS) column in a Miltenyi AutoMACS cell separator. Lin^+^ cells are retained in the column magnetically, while Lin- cells are collected as the effluent desired target population. We further enriched for Sca1-expressing cells using four iterative cycles of MACS using Sca1 antibodies conjugated with FITC and anti-FITC microbeads (Anti-Sca1 Microbead Kit, #130-092-529 Miltenyi Biotec).

To resolve the SP and non-SP (NSP) fractions within the obtained Lin^-^/Sca1^+^ cell population, the cells were stained with 5 μg/ml Hoechst 33342 (Sigma) for 90 min at 37°C, and SP cells identified on the basis of Hoechst dye exclusion. Control samples for the dye-efflux assay included Hoechst 33342 with one or more of the following efflux pump inhibitors: 10 μM fumitremorgin C (Merck), 50 μm verapamil (Sigma-Aldrich), or 5 μm reserpine (Sigma-Aldrich). To identify any potential contamination by residual Lin^+^ cells, the cell suspension was incubated with APC-conjugated streptavidin (eBioscience, #17-4317-82). Lin^-^ (APC^-^) / Sca1^+^ (FITC^+^) / SP cells were then purified by fluorescence activated cell sorting (FACS), optimized for SP cells by inclusion of a UV laser (FACSAriaII, Beckman Dickinson). Propidium iodide (PI, Sigma-Aldrich) was used at a concentration of 2 μg/mL to exclude dead cells from the final sorted population.

293FT embryonal human kidney cells (Invitrogen) were used for luciferase reporter assays and optimization of immunostaining, Lenti-X 293T cells (Clontech) for lentiviral vector packaging, and MOVAS adult mouse aortic smooth muscle cells (ATCC) for optimization of QRT-PCR assays. Neonatal cardiomyocytes were isolated from 2–3 day old mice using the Neonatal Cardiomyocyte Isolation System (Worthington Biochemical, #LK003300), including calcium- and magnesium-free HBSS, Worthington Trypsin, Worthington Soybean Trypsin Inhibitor and Worthington Purified Collagenase.

### Cell culture

Cells were cultured at 37°C in humidified air with 5% CO2. CSCs and cardiiomyocytes were cultured on collagen-coated plates, and the other cell types on routine tissue culture plasticware. During expansion and maintenance, CSCs cells were cultured in Clonal Growth Medium (CGM), adapted from Messina et al. [26]: 65% Dulbecco’s modified Eagle’s medium (DMEM) / Hams F-12 (1:1, Life Technologies); 35% Iscove's Modified Dulbecco's Medium (Life Technologies); 3.5% bovine growth serum (Merck); 100 U/ml Antibiotics-Antimycotics (Life Technologies); 2 mM L-glutamine (Life Technologies); 0.1 mM β-Mercaptoethanol (Sigma-Aldrich); 1.3% B27 media supplement (Life Technologies); 6.5 ng/ml epidermal growth factor (Peprotech); 13 ng/ml fibroblast growth factor-2 (Peprotech); 0.0005 U/ml thrombin (Roche); 0.345 ng/ml human cardiotrophin-1 (Cell Sciences). For differentiation assays, the cells were cultured in Clonal Low Proliferation Medium (CLM): IMDM; 10% BGS; 100 U/ml Antibiotics-Antimycotics; 2 mM L-glutamine: under these conditions, cell number is equal for transduced and non-transduced samples, with no significant change from day 3 to 20. All other cells were cultured in DMEM, 10% FBS, 2 mM L-glutamine, 100 U/ml Antibiotics-Antimycotics). All cells are adherent and were passaged using 0.25% Trypsin-EDTA (Life Technologies) for 5–10 min. Cell number and viability were monitored using an automated cell counter (Vi-CELL; Beckman Coulter). Unless otherwise indicated, cells were seeded at a density of 10,000 cells / cm^2^.

### Flow cytometry

The FACSAriaII for preparative cell sorting and an equivalently equipped LSRII flow cytometer (Becton Dickinson) for protein expression studies were equipped with 355 nm UV, 405 nm violet, 488 nm blue, 561 nm yellow-green and 638 nm red lasers. FlowJo version 10.0.06 (Tree Star) was used for data analysis.

### Lentiviral expression vectors

Cardiac SP cells stably overexpressing the reverse tet activator (rtTA) [42] were generated by transduction with a lentiviral rtTA-IRES-Puror vector, pNL-rtTA2s-M2 [15], encoding rtTA plus a Puromycin resistance gene (Puror) for selection. pNL-EGFP/TREPitt dU3 was from Addgene (#18660). The tetracycline-dependent vectors TRE-GATA4-IPG, TRE-MYOCD-ING, and TRE-TBX5-INR were described previously [15]. The human cardiac-enriched isoform MEF2C cDNA was obtained from Source BioScience LifeSciences (isoform 1, #OCAAo5051F05116D) and cloned into TRE-MYOCD-ING, after excising MYOCD.

Lentiviral vectors were generated using a 2^nd^ generation packaging system [74], consisting of three components, the lentiviral vector encoding the transgene(s), the packaging vector, psPAX2 (Addgene, #12260) and the envelope vector, pMD2.G (Addgene, #12259). Lentiviral particles generated using this system are encapsidated with the envelope of the vesicular stomatitis virus, the envelope glycoprotein (VSV-G) [75,76], which mediates infection of a broad range of cells independent of cell surface receptor expression [77,78]. For production, Lenti-X 293T cells seeded at 70,000 cells/cm^2^ in 10 cm^2^ dishes were transiently transfected 24 hr later using 6 μg of the transgene-encoding lentiviral vector, 4.5 μg of psPAX2 3 μg of pMD2.G, and FuGENE 6 (Promega) at a ratio of 1:3 μg:μl in serum-free DMEM. The medium was replaced with 10 ml DMEM, 10% FBS, per 10 cm^2^ dish 12 hr after transfection. The supernatant was collected 36 hr post-transfection, passed through a 0.45 μm polyethersulfone low protein-binding filter (Whatman), and stored at 4°C. Ten ml of fresh DMEM, 10% FBS, was added to each cell culture dish for collection of a consecutive supernatant 48 hr post-transfection. The viral supernatants were pooled and centrifuged on a 20% sucrose cushion in a Optima XPN100 ultracentrifuge and SW32. Ti swinging bucket rotor (Beckman Coulter) at 82,700 x g for 2 hr at 4°C. The lentiviral particle-containing pellet was dried for 2–3 min at room temperature, resuspended for 2 hr at 4°C in 260 μl PBS and vortexed every 20 min. The resuspended preparation was aliquoted and stored at -80°C.

To determine the minimal concentration of Puro that causes death of all cells after 3–5 days, a range of Puro concentrations was tested on non-transduced cells. Cells were deposited in 10 cm^2^ tissue culture dishes and transduced one day later with the rtTA-IRES-Puro^r^ lentiviral vector in 8 μg/ml polybrene (Sigma-Aldrich) in the respective cell growth media. rtTA-IRES-Puro^r^ lentiviral vectors were utilised from supernatant directly harvested during virus production, without concentration, at a volume ratio of 1:5 with cell culture medium. Two days post-transduction, 3 μg/ml Puro was added to the medium. In parallel, non-transduced cells were seeded and treated the same way. Selection with Puro was carried out for 14 days, by which stage all non-transduced cells have died. Puro-selected rtTA-transduced cardiac SP cells (CSP rtTA) were expanded and banked at -150°C for use in the later experiments.

Lentiviral vector titration was based on the expression of fluorescent reporter proteins encoded by each vector in successfully transduced cells, as determined by FACS. CSP rtTA cells were seeded at 5,000 cells / cm^2^ in 2 cm^2^ tissue culture wells of tissue culture plates and transduced one day after seeding, using 6-fold dilutions of the vector preparations from 1:25 to 1: 194,000 to in CGM plus 8 μg/ml polybrene. The tissue culture plates were centrifuged at 1,100 x g for 1 hr at 37°C and returned to the incubator. One day post-transduction, the medium was replaced by CLM containing 1 μg/ml Doxycycline (Dox, Sigma-Aldrich) to induce expression of the dual transgene (transcription factor and fluorescent reporter). Two days later, cells were harvested and analysed by FACS. Successfully transduced cells were identified by expression of fluorescent proteins encoded by the respective lentiviral vector. Only concentrations yielding < 20% fluorescent cells were taken into account for the calculation of titre, as only in this range is there a linear relationship between the percentage of fluorescent cells and the amount of lentiviral vector added [74].

### Forward programming by secondary transduction

CSP rtTA cells were seeded in CGM at 10,000 cells / cm^2^ in 9.6cm^2^ wells in 6-well plates or 0.32 cm^2^-cell culture wells in 96-well plates. One day after seeding, the mean cell number per well was calculated and the required volume of lentiviral vector preparation was determined to achieve an MOI of 100 (virus particles per cell). An MOI of 100 resulted in the highest percentage of transduced cells (range, 8 to 29%), with higher MOIs not uniformly resulting in a higher percentage of transduced cells. The required lentiviral vector preparation volume was added to fresh CGM containing 8 μg/ml polybrene, which then was used to replace the cell medium. The plates were then centrifuged, incubated for 24 hours, and treated with CLM containing 1 μg/ml Dox as above. CSP rtTA cells secondarily transduced with the transcription factor-encoding lentiviral vectors were maintained in the same vessel for 2 or 4 weeks, and the Dox-containing CLM was refreshed every 2–3 days.

### Forward programming by homogeneous transduction

CSP rtTA cells were seeded in T75 flasks at a density of 10.000 cells/cm^2^. One day after seeding, cells were transduced with concentrated lentiviral vector preparations at aMOI of 100 in fresh CGM containing 8 μg/ml polybrene. One day post-transduction, the medium was replaced with fresh CGM. The following day, the secondarily transduced CSP rtTA cells were harvested using 0.25% Trypsin-EDTA and one cell was deposited per well of a 96 well plate by preparative flow sorting (FACSAria II). The plate was maintained in the incubator and CGM was refreshed every 2–3 days. Confluency of each individual well was monitored serially, and upon reaching >70% confluency cells were harvested and replated at 10.000 cells/cm^2^ in an appropriate tissue culture vessel. Following expansion for one month, the cells were split and a portion of each culture was treated for 2 days ± Dox to confirm expression of the dual RFP and GFP cassettes. Selected cell populations expressing both reporters homogeneously (>99% prevalence) were seeded in 9.6cm^2^ well plates, as well as 96-well plates, at a density of 10,000 cells/ cm^2^. One day after seeding, cells were treated with CLM in the absence or presence of 1 μg/ml Dox, as above. After 2 weeks, cells were harvested for RNA isolation or fixed in 4% paraformaldehyde for immunostaining.

### Quantitative RT-PCR

RNA was isolated using TRIzol Reagent (Life Technologies, #15596026) in conjunction with the PureLink RNA isolation kit (Life Technologies). For 5 × 10^5^ to 5 × 10^7^ cells or ≤200 mg animal tissue, the Pure Link RNA Mini kit (Life Technologies, #12183025) was used, and for less than 5 × 10^5^ cells the Pure Link RNA Micro Scale kit (Life Technologies, #12183016). Modifications of the Pure Link RNA kit isolation procedures were as follows: Cell pellets were dissolved and tissue fragments were homogenized with a tissue homogenizer in 1 ml TRIzol (Life Technologies, #15596–026) and immediately snap-frozen on dry ice. After thawing the TRIzol sample, 0.2 ml chloroform (Sigma-Aldrich, #C2432) was added and after vigorous shaking the mixture was incubated for 10 min. The sample was centrifuged at 12,000 x g for 15 min at 4°C and then 450 μl of the colorless, upper phase containing RNA were transferred to a fresh tube. Binding of RNA to the PureLink anion exchange columns was carried out, followed by treatment with On-column PureLink DNAse (Life Technologies, #12185010). Washing steps were performed as recommended by the manufacturer, with minor modifications: for the second wash step Wash Buffer II was incubated for 5 min on the column before centrifugation. When using the Pure Link RNA Mini kit or Pure Link RNA Micro kit, RNA was eluted from the column by applying 40 or 14 μl respectively of Nuclease-free water (Qiagen, #1039498) to each and incubating for 2 min before centrifugation. Eluted RNA was stored at -80°C.

RNA samples were thawed on ice and quantitated using a NanoDrop 8000 UV-Vis spectrophotometer (Thermo Scientific). To synthesize single-stranded cDNA from total RNA, we used the High Capacity cDNA Reverse Transcription Kit (Applied Biosystems, #4368813). Briefly, a 2x reverse transcription (RT) master mix was prepared for each 100 μl-reaction. Per reaction, 50 μl of the 2x RT master mix was transferred into a tube of a MicroAmp 8-tube strip (Applied Biosystems, #PN N8010580). 1 μg of RNA was prepared in a volume of 50 μl in nuclease-free water and added to the 50 μl of 2x RT master mix, resulting in a final volume of 100 μl. The reaction mix was mixed and the 8-tube strips were sealed and centrifuged. Reverse transcription was performed in a Veriti 96 well Thermal Cycler (Applied Biosystems).

For QRT-PCR, TaqMan Low Density Arrays (Applied Biosystems) were used routinely, enabling the simultaneous analysis of 12 genes in each of 8 samples. Reactions were carried out in a volume of 100 μl, using custom-made TaqMan Array Cards pre-loaded with the TaqMan Gene Expression Assays (Table 1), and were performed on a 7900HT Fast Real-Time PCR System (Applied Biosystems).

**Table 1:** TaqMan QRT-PCR gene expression assays.

Gene	Assay Number	Gene	Assay Number
Progenitor cell markers		Cardiomyocyte markers	
*Abcg2*	Mm00496364_m1	*Actc1*	Mm01333821_m1
*Abcb1b*	Mm01324120_m1	*Cacna1c*	Mm01188822_m1
*Ly6a*	Mm00726565_s1	*Casq2*	Mm00486742_m1
**Cardiac transcription factors**		*Csrp3*	Mm00443379_m1
*Gata4*	Mm00484689_m1	*Des*	Mm00802455_m1
*Gata6*	Mm00802636_m1	*Mybpc3*	Mm00435104_m1
*Hand1*	Mm00433931_m1	*Myh6*	Mm00440359_m1
*Hand2*	Mm00439247_m1	*Myh7*	Mm01319006_g1
*Mef2a*	Mm01318991_m1	*Myl2*	Mm00440384_m1
*Mef2c*	Mm01340839_m1	*Myl3*	Mm00803032_m1
*Nkx2-5*	Mm00657783_m1	*Myl4*	Mm00440378_m1
*SRF*		*Myl7*	Mm00491655_m1
*Tbx5*	Mm00803518_m1	*Myom1*	Mm00440394_m1
*Tbx20*	Mm00451515_m1	*Myom2*	Mm00500665_m1
**Smooth muscle markers**		*Myoz2*	Mm00469639_m1
*Cnn1*	Mm00487032_m1	*Nppa*	Mm01255748_g1
*Myh11*	Mm00443013_m1	*Nppb*	Mm01255770_g1
**Loading controls**		*Pln*	Mm00452263_m1
*Hmbs*	Mm01143545_m1	*Ryr2*	Mm00465877_m1
*Ubc*	Mm01201237_m1	*Smyd1*	Mm00477663_m1
		*Srl*	Mm00614763_m1
**Human assays**		*Tnnc1*	Mm00437115_g1
*GATA4*	Hs00171403_m1	*Tnni3*	Mm00437164_m1
*MEF2C*	Hs00231149_m1	*Tnnt1*	Mm00449089_m1
*MYOCD*	Hs00538076_m1	*Tnnt2*	Mm01290252_g1
*TBX5*	Hs01052563_m1	*Tpm1*	Mm00600378_m1
		*Ttn*	Mm00621005_m1

All assays were carried out in TLDA format except for SRF, which was analysed individually.

### Western blotting and immunocytochemistry

Nuclear and cytosolic protein fractions of homogenized cells were prepared using Nuclear/Cytosol Fractionation kits (Biovision, USA#K266-100) as described in the manufacturer’s instructions. The recommended volume of all lysis buffers was doubled when the cell number per sample exceeded 4 x 10^6^ cells. To validate no or minimal contamination of the nuclear fraction with cytosolic proteins or vice versa, we analysed expression of Histone H1 as a nuclear marker and GAPDH or HSP90 as cytosolic markers. Histone H1 is part of the histone complex around which the DNA is tightly wrapped and which together with the DNA forms the basic unit of chromatin, the nucleosome [79]. GAPDH serves an important metabolic function in eukaryotic cells as it catalyses the breakdown of glucose during glycolysis, a process which takes place in the cytosol [80]. HSP90 is a molecular chaperone, which guides efficient protein folding of many newly synthesized proteins in the cytosol [81]. Total protein lysates were isolated using 10 x cell pellet volume of RIPA buffer (Sigma, #R0278) supplemented with 1x Phosphatase (Roche Applied Biosystems, #04906845001) and 1x Protease Inhibitor Cocktail (Roche Applied Biosystems, #04693159001). The protein concentration was determined using the Pierce BCA Protein Assay Kit (Thermo Scientific, #23225).

Protein samples were prepared by denaturation for 10 min at 95°C in 4x Sample Buffer. Proteins were electrophoresed on 10 or 12% separating gels with 4% stacking gels in 1x Running Buffer at 120 V for up to 2 hr by vertical sodium dodecyl sulfate polyacrylamide gel electrophoresis (SDS-PAGE) in a Mini-PROTEAN Tetra cell (BioRad). Proteins were then transferred to Hybond ECL nitrocellulose membranes (GE Healthcare, #RPN3032D) at 110 V for 1.5 hr in 1x Transfer Buffer in a Mini Trans-Blot module (BioRad).

Following blocking for 1 hr in Blocking Solution consisting of 5% semi-skimmed milk in PBS-0.1% Tween-20 (Sigma, #P5927) at RT, the respective primary antibody diluted in Blocking Solution (Table 2) was incubated overnight at 4°C. Subsequently, the membrane was washed 6 times in PBS-0.1%Tween-20 for 10 min each and incubated with the appropriate horseradish peroxidase (HRP)—conjugated secondary antibody (Table 2) diluted 1:1000 in 5% semi-skimmed milk in PBS-0.1% Tween-20. The membrane was washed as before and bound HRP-conjugated secondary antibodies were detected using enzyme linked chemiluminescence (ECL) detection reagents (GE Healthcare, #RPN2209) and Amersham Hyperfilm ECL (GE Healthcare, #28-9068-40).

**Table 2:** Antibodies.

Antigen target	Species raised	Isotype	Application used (conc in μg/ml)	Company	Product Code
**Cardiac transcription factors and co-factors**					
Baf60c	Rabbit	IgG	WB (0.6), IF (2.9)	ProtTech	12838-1-AP
Gata4	Mouse	IgG2b	WB (1), IF (10)	R&D	MAB2606
Hand2	Goat	IgG	WB (1), IF (2)	R&D	AF3876
Mef2a	Rabbit	IgG	WB (1–3), IF (5–15)	Abcam	ab76063
Mef2c	Rabbit	IgG	WB (0.2), IF (0.84)	Cell Signaling	5030
Myocd	Mouse	Serum	WB (1–10), IF (4–40)	Abcam	ab22073
Nkx2-5	Goat	IgG	WB (1), IF (1)	Santa Cruz	sc8697
Tbx5	Mouse	IgG1	WB (4), IF (2.5)	Abnova	HRP0006910-M01
Tbx20	Rabbit	IgG	WB (0.5), IF (1)	Orbigen	ARP-PAB11248
**Nuclear fraction loading controls**					
H1 + core proteins	Mouse	IgG2b	WB (7.5)	Millipore	MABE71
**Cytosolic fraction loading controls**					
GAPDH	Rabbit	IgG	WB (dilution 1/1000)	Cell Signaling	2118
Hsp90	Rabbit	IgG	WB (dilution 1/1000)	Cell Signaling	4877
**Whole cell loading control**					
β-actin	Rabbit	IgG	WB (dilution 1/1000)	Cell Signaling	4967
**Cardiac structural proteins**					
α-cardiac actin	Mouse	IgG1	IF (1.25)	Sigma	A9357
sarc MyHC	Mouse	IgG2a	IF (1.25)	R&D	MAB4470
ANF	Rabbit	IgG	IF (4)	Millipore	ab5490
Tpm1	Mouse	IgG1	IF (12)	Sigma	T2780
cTnT	Mouse	IgG1	IF (1)	Santa Cruz	sc52284
**Smooth muscle proteins**					
Cnn1	Rabbit	IgG	IF (0.5)	Epitomics	1806–1
Myh11	Rabbit	IgG	IF (1.25)	Biomedical Technologies	BT562
**Isotype controls**					
IgG	Goat	IgG	As required	Vector Labs	I-5000
IgG	Mouse	IgG	As required	Vector Labs	I-2000
IgG	Rabbit	IgG	As required	Vector Labs	I-1000
IgG1	Mouse	IgG1	As required	Sigma	M5284
IgG2A	Mouse	IgG2a	As required	Abcam	ab18414
**Secondary antibodies**					
Anti-mouse IgG, HRP	Horse	--	WB (dilution 1/3000)	Cell Signaling	7076
Anti-rabbit IgG, HRP	Goat	--	WB (0.1)	Abcam	ab6112
Anti-goat IgG, HRP	Rabbit	--	WB (0.2)	Abcam	ab5755
Anti-mouse IgG, Alexa 488	Goat	--	IF (2)	Cell Signaling	4408
Anti-rabbit IgG, Alexa 488	Goat	--	IF (2)	Cell Signaling	4412
Anti-goat IgG, Alexa 488	Chicken	--	IF (2)	Life Technologies	A21467
Anti-mouse IgG, Alexa 555	Goat	--	IF (2)	Cell Signaling	4409
Anti-rabbit IgG, Alexa 555	Goat	--	IF (2)	Cell Signaling	4413
Anti-goat IgG, Alexa 555	Donkey	--	IF (2)	Life Technologies	A11057
Anti-mouse IgG, Alexa 647	Donkey	--	IF (3)	Jackson Immunolabs	715-495-150
Anti-goat IgG, Alexa 647	Donkey	--	IF (3)	Jackson Immunolabs	705-606-147
Anti-rabbit IgG, Alexa 647	Donkey	--	IF (3)	Jackson Immunolabs	711-606-152

IF, immunofluorescence; HRP, horseradish peroxidase; WB, Western blot. All secondary antibodies were F(ab')2 fragments excepting horse anti-mouse IgG, which was intact IgG.

Cells cultured in 96-well plates were washed with PBS and then fixed in 100 μl per well of 4% paraformaldehyde in PBS (pH 7.4, Sigma, #P6148) for 15 min at RT. Subsequently, cells were washed 3 times with PBS and 100 μl per well blocking buffer was added, which was incubated for 1 hr at RT with constant shaking of the plate (indicate rpm and apparatus). Each primary antibody, diluted in blocking buffer as per Table 2, was incubated overnight at 4°C, followed by 3 wash steps with 0.02% Triton-X100 (Sigma, #X100) in PBS, and 3 wash steps in PBS. Species-specific secondary antibodies, conjugated with Alexa fluorophores, were diluted as in Table 2 and incubated on the cells for 1 hr at RT. The same wash steps described above were carried out and then nuclei were counter-stained with 4 μg/ml 4',6-diamidino-2-phenylindole (DAPI; Sigma, #D9542) in PBS for 2 min at RT. DAPI solution was removed, fresh PBS was added, and plates were covered in aluminium foil and stored at 4°C until microscopic analysis. Image acquisition was performed on an Axio Observer.Z1 (Zeiss) inverted fluorescence microscope at 20 x magnification using AxioVision v 4.8 (Zeiss).

To validate antibody specificity, ee transfected 293FT cells with expression vectors encoding murine cardiac transcription factors and used these ectopic factor-overexpressing cells and their GFP control plasmid-transfected counterparts as positive and negative controls for antibody testing. 293FT cells were seeded at a density of 20,000 cells / cm^2^ in 9.6 cm^2^-wells (60-well plates) for protein lysate extraction or 0.32 cm^2^-wells (96-well plates) for immunostaining and were transfected with 3 μg of the transcription factor-encoding plasmids using 6 μl Fugene 6 for 12 hours. Two days post-transfection cells were harvested from 9.6 cm^2^-wells or fixed in situ using 4% PFA in 0.32 cm^2^-wells.

### High content image analysis

Plates were scanned on a Cellomics ArrayScan VTI high content screening instrument using ArrayScan image acquisition and analysis software (Thermo Fisher). Using its automated Zeiss Observer Z1 epifluorescence microscope, 49 images completely covering each 0.32 cm^2^-well were acquired with suitable filter sets at 10× magnification. Fluorescence intensity was recorded in channels 1–4 using the filter sets XF93 Hoechst (DAPI), XF93 FITC (GFP), XF93-Cy5 (Alexa 647), and XF32-TRITC^sensitive^ (dsRed), respectively.

In each well, between 6200 and 14700 cells were detected, depending on the cell line analysed and the respective ectopic transcription factor combination with which the population was transduced. Each treatment was tested in duplicate wells, except where indicated.

Two different algorithms modified from Thermo Fisher were utilised to quantitate the fraction of transduced cells staining for cardiac marker proteins. One common feature to all protocols is the first step of identifying every cell per well and classifying them as objects, which are then analysed for fluorescence intensity in the other channels. Object identification was either based on DAPI expression in the nucleus or expression of nuclear-localised dsRed, where appropriate, in all cells of a given population. Following object identification, an area or interest relative to the objects was defined, within this area the intensity of signal for the specific markers was measured. A threshold was based on fluorescent intensity in the negative control. For endogenous cardiac markers in cells transduced with ectopic transcription factors, the negative control was immunostaining of GFP control virus-transduced cells, stained in parallel. Cells were classified as positive or negative for fluorescent marker expression or antibody staining depending on their fluorescence intensity above or below the threshold, respectively. Due to potential differences in background fluorescence intensity in different cell lines, an individual threshold was used for each analysed cell line. For GFP and dsRed expression, autofluorescence of untransduced cells was used as the negative control. Further details of the procedures for image analysis are indicated in Table 3.

**Table 3:** High-content image analysis of cardiac protein induction.

Cell population		Image processing	
	**Step 1 (TA)**	**Step 2 (TA)**	**Step 3 (TA)**
Heterogeneously transduced cells stained with ANF, Ryr2	Object identification by DAPI, fitting of DAPI ring, creation of signal ring, by expansion of DAPI ring by specific factor	Identification of transduced cells based on average fluorescence intensity in GFP and dsRed channel within signal ring	Identification of cardiac marker-stained cells based on total fluorescence intensity in A647 channel within signal ring
	**Step 1 (TA)**	**Step 2 (TA)**	**Step 3 (SD)**
Heterogeneously transduced cells stained with α-cardiac actin, sarc MyHC	Object identification by DAPI, fitting of DAPI ring, creation of signal ring, by expansion of DAPI ring by specific factor	Identification of transduced cells based on average fluorescence intensity of GFP and dsRed channel within signal ring	Identification of cardiac marker-stained cells based on fluorescence spot intensity across the whole cell in A647 channel
	**Step 1 (TA)**	**Step 2 (TA)**	**Step 3 (TA)**
Uniformly MyoT-transduced cells stained with α-cardiac actin, sarc MyHC, ANP, Tpm1, or cTnT	Object identification by dsRed^nuc^, fitting of dsRed^nuc^ ring, creation of signal ring, by expansion of dsRednuc ring by specific factor	Identification of transduced cells based on average fluorescence intensity of GFP and dsRed channel within signal ring	Identification of cardiac-marker stained cells based on average fluorescence intensity in A647 channel within signal ring

Transduced cells were identified as shown. For further discrimination between GFP^nuc^ and GFP^perox^, cells identified as GFP^+^ were individually scored by visual inspection as GFP^nuc+^ / GFP^perox+^, GFP^nuc—^/ GFP^perox+,^ or GFP^nuc+^ / GFP^perox-^. TA, Target Activation algorithm; SD, Spot Detection algorithm.

Successfully transduced cells were identified based on expression of GFP^nuc^ (for MEF2C and MYOCD) and GFP^perox^ (for GATA4), or dsRed^nuc^ (for TBX5). Cardiac marker protein staining was performed for α-cardiac actin, ANF, sarcomeric (sarc) MHC, Ryr2, Tpm1, or cTnT using respective specific primary antibodies and species-specific Alexa647-conjugated secondary antibodies (Table 1).

### Statistics

Post-hoc tests were performed using Prism v. 6 (Graphpad). All results are expressed as the mean ± standard deviation (SD). To test significance in differences of multiple genes among different samples, one-way ANOVA was performed, followed by the Tukey-Kramer multiple comparisons post-hoc test. In studies comparing a particular gene in ectopic transcription factor-expressing cells versus GFP control cells, induction of each gene was tested individually for significance using the unpaired, two-tailed Student’s t test. Differences of p < 0.05 were considered statistically significant.

## Supporting Information

S1 Fig Validation of ectopic cardiac transcription factor expression from lentiviral constructs. A: Immunocytochemistry. 293FT cells were transfected with plamids encoding the respective cardiac transcription factor plus GFP, versus the GFP control plasmid, then were analysed as in Fig 4B. Successfully transfected cells were resolved by co-expression of GFP in all cases except for TBX20, for which cells were identified by co-staining for the C-terminal V5 tag of the ectopic protein. B: Western blotting. Primary antibody binding was visualized using secondary antibodies conjugated with horseradish peroxidase (HRP) and chemiluminescence. To avoid conflicts with the molecular weight of the respective transcription factors, different loading controls were chosen: Gapdh for Baf60c, Tbx5 and Tbx20; α-tubulin for Gata4; and heat shock protein 90 (Hsp90) for Hand2, Mef2a, Mef2c, and Nkx2-5.
(PDF)

S2 Fig Optimizing the bicistronic lentiviral system. A: Viral titer. CSP rtTA cells transduced with the vectors shown were treated with Dox for 2 days and scored on the basis of fluorescent reporter expression. Data are the mean ± SD for 3 samples. B-D: Dox concentration. B: Representative phase contrast and epifluorescence images. C: Mean ± SD for 3 samples. *, p < 0.05 ± Dox. D: Western blot, showing Dox-dependent induction of ectopic GATA4.
(PDF)

S3 Fig Stemness genes and endogenous transcription factors in CSCs homogeneously transduced with *MYOCD* and *TBX5*. Data are the mean ± SD of three independent experiments. *, p < 0.05 versus day 0. A: Down-regulation of genes for Sca1 and the SP phenotype. B: Lack of endogenous transcription factor induction.
(PDF)
