# Supplementary figures and images for: Forward Programming of Cardiac Stem Cells by Homogeneous Transduction with MYOCD plus TBX5
Source: PLoS One. 2015 Jun 5;10(6):e0125384. doi: 10.1371/journal.pone.0125384 (PMC4457652; doi:10.1371/journal.pone.0125384)

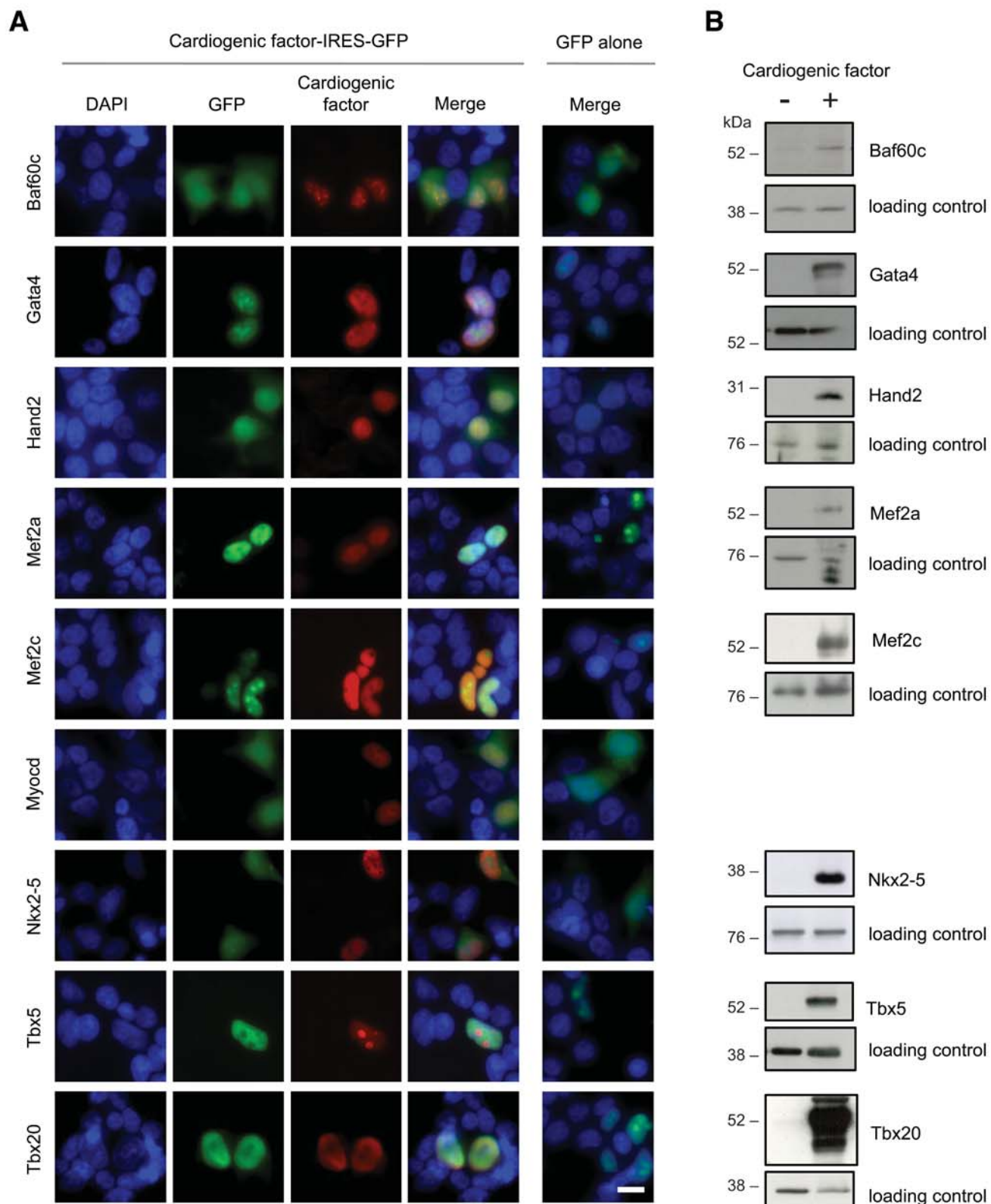

Supplement: S1 Fig — A: Immunocytochemistry. 293FT cells were transfected with plamids encoding the respective cardiac transcription factor plus GFP, versus the GFP control plasmid, then were analysed as in Fig 4B. Successfully transfected cells were resolved by co-expression of GFP in all cases except for TBX20, for which cells were identified by co-staining for the C-terminal V5 tag of the ectopic protein. B: Western blotting. Primary antibody binding was visualized using secondary antibodies conjugated with horseradish peroxidase (HRP) and chemiluminescence. To avoid conflicts with the molecular weight of the respective transcription factors, different loading controls were chosen: Gapdh for Baf60c, Tbx5 and Tbx20; α-tubulin for Gata4; and heat shock protein 90 (Hsp90) for Hand2, Mef2a, Mef2c, and Nkx2-5. (PDF) [file pone.0125384.s001.pdf]

**A**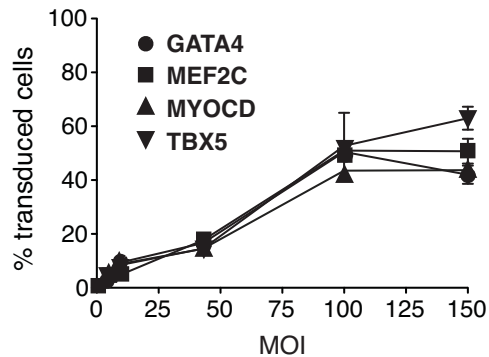**B**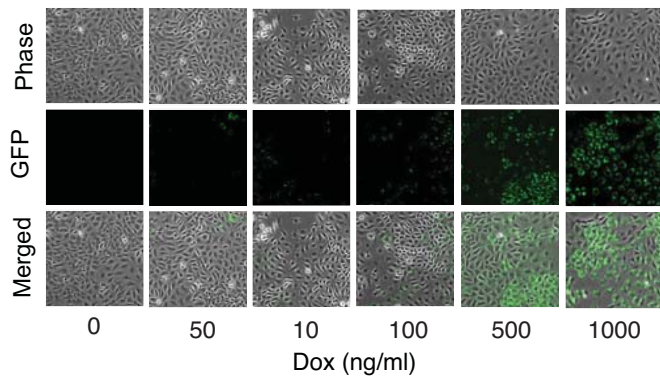**C**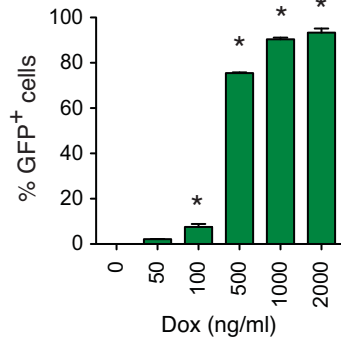**D**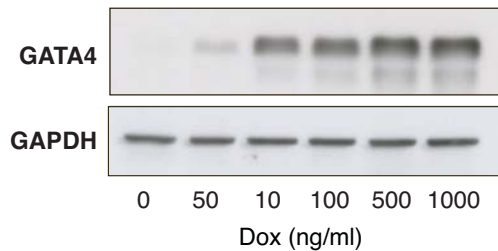

Supplement: S2 Fig — A: Viral titer. CSP rtTA cells transduced with the vectors shown were treated with Dox for 2 days and scored on the basis of fluorescent reporter expression. Data are the mean ± SD for 3 samples. B-D: Dox concentration. B: Representative phase contrast and epifluorescence images. C: Mean ± SD for 3 samples. *, p < 0.05 ± Dox. D: Western blot, showing Dox-dependent induction of ectopic GATA4. (PDF) [file pone.0125384.s002.pdf]

**A**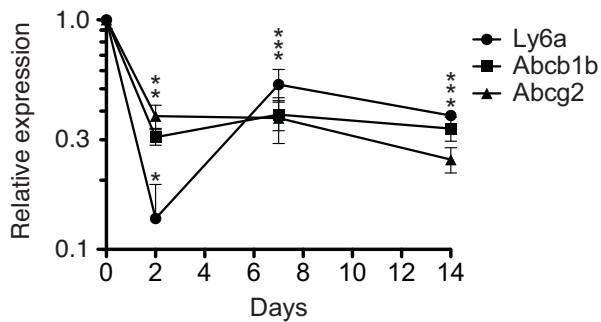**B**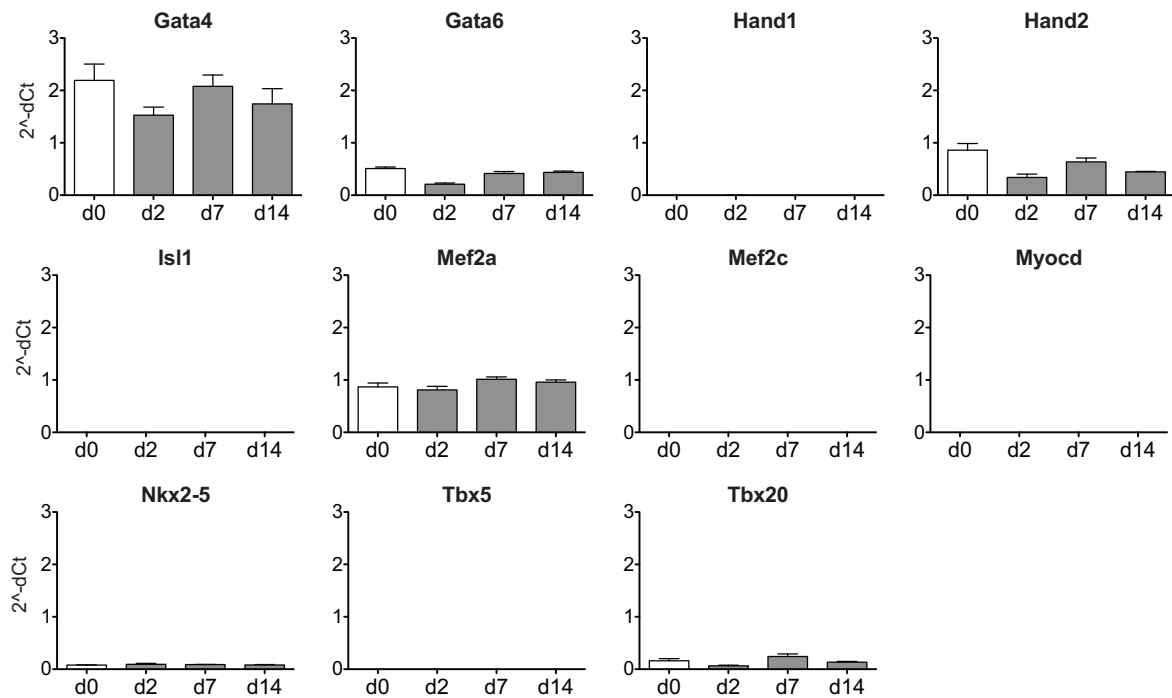

Supplement: S3 Fig — Data are the mean ± SD of three independent experiments. *, p < 0.05 versus day 0. A: Down-regulation of genes for Sca1 and the SP phenotype. B: Lack of endogenous transcription factor induction. (PDF) [file pone.0125384.s003.pdf]
